# Ultrasmall Copper-Based Nanozyme Eye Drops for Effective Antioxidative Therapy of Ocular Surface Diseases

**DOI:** 10.1021/acsomega.5c00103

**Published:** 2025-06-16

**Authors:** Rui Qiao, Liyuan Yang, Shu Zhang, Meiling Qian, Yu Lu, Huiling Bai, Qin Liu

**Affiliations:** † 381940 Gansu University of Chinese Medicine, Lanzhou, Gansu 730000, P. R. China; ‡ The First People’s Hospital of Lanzhou City, Lanzhou, Gansu 730050, P. R. China; § Department of Ophthalmology, 91589 Gansu Provincial Hospital, Lanzhou, Gansu 730000, P. R. China; ∥ Lanzhou Aier Eye Hospital, Lanzhou, Gansu 730099, P. R. China

## Abstract

Developing an effective strategy to mitigate the excessive production of reactive oxygen species (ROS) caused by diverse factors is crucial for preventing ocular surface diseases. However, due to the inherent characteristics of ocular barriers, the therapeutic efficacy of conventional eye drops remains unsatisfactory. Copper-based nanozymes are known for their enzyme-mimetic ROS scavenging abilities. In this study, we report a simple, ecofriendly, one-step synthesis of ultrasmall copper Cu_5.4_O nanoparticles (NPs) as antioxidant and anti-inflammatory nanozymes in eye drop formulations. The Cu_5.4_O NPs showed strong hydrogen peroxide (H_2_O_2_) scavenging ability and reduced intracellular ROS in corneal cells *in vitro*. *In vitro* hemolysis studies and *in vivo* assessments of ocular biocompatibility confirmed that Cu_5.4_O NP eye drops are safe for application as nanomedicines in ophthalmic formulations. Moreover, in the H_2_O_2_-induced corneal oxidative damage model in mice, Cu_5.4_O NP eye drops effectively scavenged ROS and reduced inflammation, thereby providing protection against oxidative damage and facilitating epithelial regeneration. Collectively, the capabilities of Cu_5.4_O NP-based eye drops offer valuable insights into developing new treatment strategies for ROS-mediated corneal diseases and support the use of nanomaterials in clinical applications.

## Graphical abstract

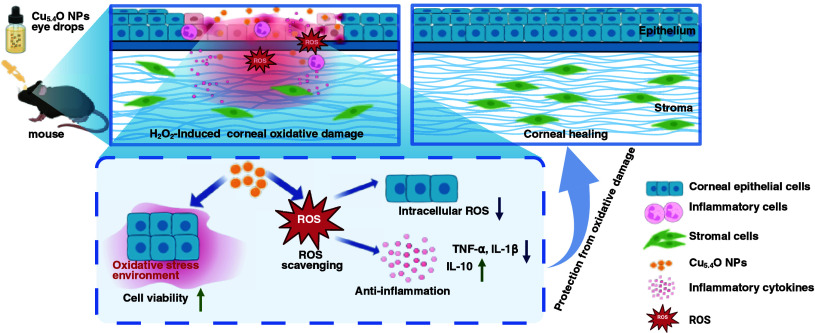

## 1 Introduction

Reactive oxygen species (ROS) are caused during standard physiological functions in healthy individuals and are usually regulated by the organism antioxidant defense mechanisms to prevent oxidative stress damage. Nevertheless, when the product level of ROS exceeds the antioxidant defense capacity of cells, oxidative stress ensues. This phenomenon plays a vital function in the pathogenesis of various disorders.^1,2^ In ophthalmology, elevated and dysregulated ROS levels are linked to various ocular conditions, including dry eye syndrome, keratitis, uveitis, cataracts, glaucoma, age-related macular degeneration, and retinopathy of prematurity.^3^ Given the substantial impact of oxidative stress in these conditions, maintaining ROS balance on the ocular surface has become a possible treatment strategy for the prevention and management of ROS-related ocular disorders.

As an enzyme-like nanomaterial, antioxidant nanoenzymes have garnered considerable interest across diverse medicinal domains owing to their multifunctionality.^4^ Copper, an essential micronutrient in the human body, is a crucial constituent of various natural enzymes and facilitates numerous vital biological processes.^5^ Recent research has emphasized the capability of copper-based nanozymes in scavenging ROS. Zeng et al. demonstrated that multienzyme-mimicking Au@Cu_2_O heterostructures possess comprehensive antioxidant capacity against ROS.^6^ Chen et al. created a two-dimensional copper-based antioxidant nanozyme that effectively eliminated ROS and promoted healing in chronic diabetic wounds.^7^ In addition, Chen et al. demonstrated that a Cu SAs/CN nanozyme, which exhibits robust ascorbate peroxidase-like activity and good biocompatibility, efficiently protected cells exposed to hydrogen peroxide (H_2_O_2_) from oxidative damage *in vitro*.^8^ These findings indicate that Cu-based nanozymes may alleviate oxidative stress and safeguard the cornea from oxidative injury.

Topical administration of ocular drops is the prevalent and most convenient approach for delivering medications to the eye, whether aimed at the anterior ocular tissues or treating conditions in the posterior parts of the eye.^9^ The efficacy of eye drops is restricted by the densely arranged cellular walls of the eye, which impede the infiltration of bigger nanoparticles (NPs).^9^ For example, antioxidant eye drops containing agents like vitamin B12 or visomitin have demonstrated limited success in preventing ocular diseases, mainly due to issues with poor absorption, rapid metabolic breakdown, and irreversible consumption.^10–12^ Recent advances in nanomaterials offer promising strategies for overcoming the blood–retina barrier, thanks to their nanoscale dimensions (under 100 nm) and adaptable physicochemical properties, which support effective drug delivery.^13^ Notably, NPs with ultrasmall dimensions (<10 nm) are particularly promising for increased drug delivery, effective ROS removal, and improved therapeutic efficiency.^14,15^ Chen et al. demonstrated that among three types of Ce-MOFs with distinct particle sizes (213 nm, 36 nm, and ultrasmall at 2 nm) the ultrasmall exhibited superior ROS scavenging ability and antioxidant capacity, lower cytotoxicity, and improved ocular penetration.^16^ Liu et al. successfully synthesized Cu_5.4_O NPs with uniform ultrasmall sizes (approximately 4.5 nm), which exhibited multiple enzyme-like activities. The Cu_5_._4_O NPs have shown exceptional effectiveness in eliminating ROS at notably low dosage, proving effective in the treatment of ROS-associated disorders such as acute liver injury, acute kidney injury, and chronic wound healing.^17^ In addition, Tang et al. demonstrated that small organic molecule-based NPs (2TT-mC_6_B@Cu_5.4_ONPs) possess superior abilities to scavenge ROS and produce O_2_ synergistically, thereby relieving inflammation, alleviating hypoxia conditions, and promoting deep penetration into chronic wound tissues.^18^ Based on these findings, we hypothesized that developing ultrasmall copper-based nanozymes could provide strong antioxidant activity for treating ROS-related ocular diseases.

This study synthesized ultrasmall Cu_5.4_O NPs with good biosafety and ROS-scavenging capabilities using a straightforward and environmentally friendly approach, aimed at therapeutic applications for ROS-related ocular diseases (Scheme 1). Cu_5.4_O NPs exhibited an ultrasmall particle size and excellent *in vitro* stability. Additionally, they demonstrated strong biocompatibility and antioxidant ability, low hemolytic activity *in vitro*, and high safety in corneal biocompatibility assessments *in vivo*. In the H_2_O_2_-induced corneal oxidative damage model in mice, Cu_5.4_O NP eye drops effectively protected corneal tissue by reducing ROS levels and inflammation, thus promoting corneal regeneration. This study emphasizes the efficacy of Cu_5.4_O NPs as a viable therapeutic strategy for ROS-related ocular diseases.

**Scheme 1:**
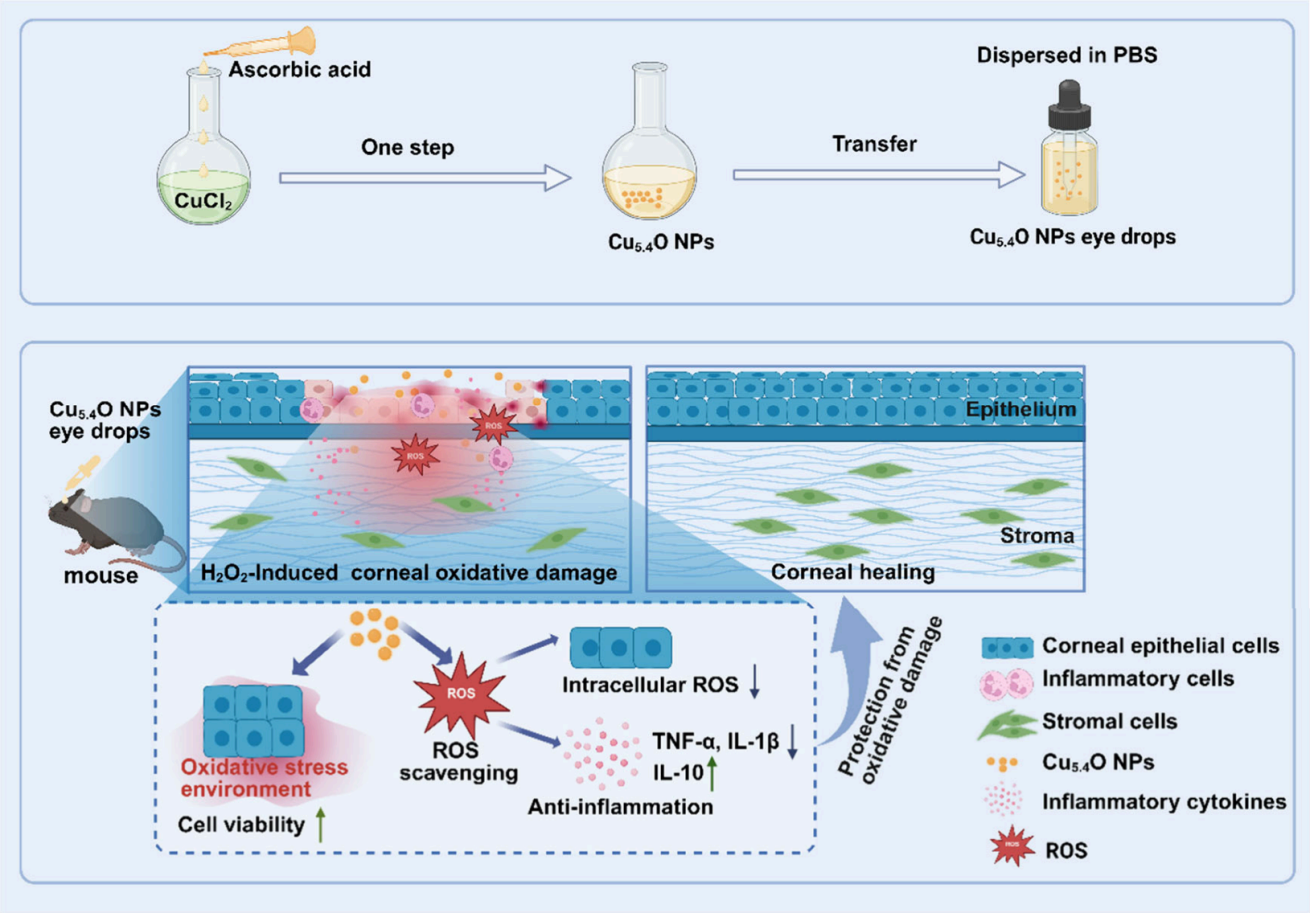
Synthesis of Cu_5.4_O NP Eye Drops and Therapeutic Mechanism in H_2_O_2_-Induced Corneal Damage Treatment^a^

a Top: The preparation diagram of ultrasmall size Cu_5.4_O NP eye drops was developed using a one-step method. Bottom: following the administration of the eye drops (mouse model of corneal injury induced by H_2_O_2_), Cu_5.4_O NPs maintain corneal epithelial cell viability, scavenge intracellular ROS production, and reduce inflammation, thereby promoting corneal healing. Schematic illustration was created using BioRender (www.biorender.com).

## 2 Materials and Methods

### 2.1 Preparation of Cu_5.4_O NPs

The Cu_5.4_O NPs were synthesized following a previously documented procedure.^17^ In brief, 0.0672 g of anhydrous CuCl_2_ was dissolved in 50 mL of deionized water and agitated for 10 min until completely dissolved, resulting in a 10 mM CuCl_2_ solution. The flask was subsequently positioned into an oil bath at 80 °C. An ascorbic acid solution was gradually introduced to the CuCl_2_ solution using a drip funnel. Following the addition, the mixture was stirred overnight at 80 °C. Then, the mixture solution was subsequently centrifuged at 8,000 rpm for 15 min, discarding the precipitate and preserving the supernatant. Finally, Cu_5.4_O NPs were obtained by dialyzing the supernatant in a dialysis bag (Mw cutoff: 3500 Da) for 2 days.

### 2.2 Characterization of Cu_5.4_O NPs

The morphology of Cu_5.4_O NPs was observed by transmission electron microscopy (TEM). Each particle size was quantified using ImageJ Software with a minimum analysis of 500 particles per sample. The hydrated particle size and zeta potential of the Cu_5.4_O NPs were analyzed with a Zetasizer Nano S90. Additionally, the NPs were characterized by Fourier transform infrared (FTIR) spectroscopy, spanning the range from 400 to 4000 cm^–1^.

### 2.3 H_2_O_2_ Scavenging Activity of Cu_5.4_O NPs

The H_2_O_2_ assay kit (Beyotime, Jiangsu, China) was employed to assess the H_2_O_2_ scavenging efficacy of Cu_5.4_O NPs. Various concentrations of Cu_5.4_O NPs (0.0625–2 μg mL^–1^) were formulated. Subsequently, an equivalent volume of H_2_O_2_ was introduced and incubated for 30 min in the dark. Following centrifugation at 12000 rpm for 5 min, the H_2_O_2_ concentration in the obtained supernatant was assessed by reacting it with an H_2_O_2_ assay buffer for 30 min. The optical density (OD) was measured by using a microplate reader (Molecular Devices Spectramax M5) at 560 nm, and the H_2_O_2_ scavenging rate was computed.

### 2.4 Cell Culture

Human corneal epithelial cells (HCECs) were sourced from the Lanzhou University Second Hospital in Gansu, China. HCECs were cultured in Dulbecco’s Modified Eagle Medium/F-12 (DMEM/F-12) (Procell, Wuhan, China) containing 10% fetal bovine serum with 1% penicillin–streptomycin.

### 2.5 Cell Viability

1 × 10^4^ HCECs were seeded per well in a 96-well plate. Then the cells were cultured overnight until the cells attached to the wall. Subsequently, varying concentrations of Cu_5.4_O NPs (0–5 μg mL^–1^) were administered to each group for incubation periods of 24 or 48 h. Finally, cells were incubated with a Cell Counting Kit-8 (CCK-8) working solution (Dojindo, Kumamoto, Japan) for 1 h. Cell viability was evaluated by the cell absorbance measured by a microplate reader following each treatment.

### 2.6 Live/Dead Assay

HCECs were inoculated in 96-well plates and cultured for 24 h, allowing the cells to attach to the walls. Cells were incubated with H_2_O_2_ (250 μM), Cu_5.4_O NPs (1 μg mL^–1^), or H_2_O_2_ + Cu_5.4_O NPs for 24 h, respectively. Upon completion of coincubation, the plate was meticulously rinsed three times with PBS solution. Cells were stained with a Calcein-AM/PI Double Staining kit (Beyotime, Jiangsu, China) following the manufacturer’s protocol. The cells were examined and captured using a confocal laser scanning microscope (CLSM, Zeiss LSM780, Germany).

### 2.7 *In Vitro* ROS Scavenging of Cu_5.4_O NPs in HCECs

HCECs were inoculated in 24-well plates and cultured for 24 h and then incubated with H_2_O_2_ (200 μM) for 2 h. Subsequently, the concentrations of Cu_5.4_O NPs (1 μg mL^–1^) were added into the cell culture medium and cocultured for 24 h. Subsequently, cells were rinsed with PBS 3 times to remove the free Cu_5.4_O NPs. Then, 10 μM DCFH-DA was added to the cells and cocultured for 30 min avoiding light. Finally, the unloaded DCFH-DA probe was removed, and images were captured using CLSM. The ROS fluorescence intensity of each fluorogram was obtained semiquantitatively by ImageJ software.

### 2.8 *In Vivo* Biocompatibility Evaluation of Cu_5.4_O NPs

Healthy male C57BL/6 mice (6–8 weeks old, 20–25 g) without eye illness were chosen for animal tests according to the protocols sanctioned by the laboratory animal welfare and ethics council of the Army Medical University (AMUWEC20237056). All mice employed in the animal research were provided with sufficient food and water and kept in light/dark circulation at room temperature.

To evaluate the *in vivo* biocompatibility of Cu_5.4_O NPs, mice were administered a single intravenous dose of 100 μg kg^–1^ of Cu_5.4_O NPs (equivalent to 2 μg mL^–1^ for a 20 g mouse with approximately 1 mL of blood). This dose was 10 times higher than that used in the mouse corneal oxidative damage model (20 μL of 2 μg mL^–1^ solution per eye, corresponding to approximately 0.1 μg mL^–1^ in the corneal tissue). The control group mice were administered PBS. Following a 24 h injection period, blood samples were obtained for comprehensive blood panel analysis including white blood cells (WBCs), red blood cells (RBCs), and platelets (PTLs) and serum biochemistry testing, indicators of liver function and kidney function. The mice were euthanized to procure vital organs (including the heart, liver, spleen, lung, and kidney) for hematoxylin and eosin (H&E) staining and histological examination.

### 2.9 Hemolysis Assay

Fresh whole blood from the eyes of C57BL/6 mice (6–8 weeks old, 20–25 g) was collected for the hemolysis assay. Erythrocytes were obtained using centrifugation at 2000 rpm for 10 min and subsequently washed repeatedly with normal saline until the supernatant was colorless and transparent. Subsequently, 1 mL of red blood cell precipitate was combined with 3.67 mL of physiological saline. 100 μL of red blood cell solution was introduced into a 1.5 mL eppendorf tube and incubated with different concentrations (0.125, 0.25, 0.5, 1, and 5 μg mL^–1^) of Cu_5.4_O NP solution at 37 °C for 2 h. Normal saline was used as the negative control, whereas ultrapure water functioned as the positive control group. At last, the solution was imaged following centrifugation of the cells. The absorbance of hemoglobin at 540 nm was quantified. Hemolysis ratio (%) = (AM – AN)/(AW – AN) × 100%, where AM, AN, and AW denote the absorbance of erythrocytes subjected to Cu_5.4_O NPs, physiological saline, and ultrapure water, respectively.

### 2.10 Animal Experiments

A single dose of H_2_O_2_ was administered to induce corneal oxidative stress injury in mice, with minor modifications to the method described in the literature.^19^ H_2_O_2_-induced C57BL/6 mice (6–8 weeks old, 20–25 g) corneal oxidative damage model: Initially, before the trial, healthy mice devoid of ocular surface abnormalities were chosen. The mice were subjected to general anesthesia induced by pentobarbital sodium. The mice were subsequently allocated into three groups and administered PBS (Control), H_2_O_2_ (20 μL, 1M), or H_2_O_2_ + Cu_5.4_O NP solution (20 μL, 2 μg mL^–1^) on the ocular surface for a duration of 24 h. After 24 h, the mice were euthanized, and the ocular globes were fixed with paraformaldehyde, dehydrated with gradient alcohol, then embedded and sliced for subsequent H&E staining and Masson trichrome staining. The dyed corneal tissues were subsequently examined using an optical microscope.

### 2.11 Histopathological Analysis

Following the euthanasia of the mice via anesthetic overdose, the eyes and adnexa were preserved in FAS eyeball fixative (Servicebio, Wuhan, China), subsequently embedded in paraffin, sectioned sagittally (5 μm thick), and maintained at ambient temperature. Ocular sections were subjected to histological analysis. The cell count in the epithelial layer of corneal tissue, as well as the thickness of the corneal epithelial layer and corneal stroma, were assessed utilizing ImageJ software.

### 2.12 Dihydroethidium (DHE) Fluorescence Staining

Following the euthanasia of the mice via anesthetic overdose, the intact eyeball, encompassing the upper and lower eyelids, was imbedded in an ideal cutting temperature compound. Subsequently, we quantified ROS production utilizing a DHE Assay Kit (Beyotime, Jiangsu, China). Fluorescent probe loading involves incubating eye sections in a suitable solution containing 2.5 μM of DHE at 37 °C for approximately 20 min, followed by adequate washing, with DAPI employed for nuclear counterstaining. Subsequent to covering, they were examined using a CLSM. The fluorescence intensity was assessed by using ImageJ software.

### 2.13 Enzyme-Linked Immunosorbent Assays (ELISAs)

Following the euthanasia of the mice via anesthetic overdose, corneal tissues from each group were procured by using a corneal trephine. Corneal homogenates were produced by following the methods of various experiments. The expression levels of interleukin-10 (IL-10), interleukin-1beta (IL-1β), and tumor necrosis factor-α (TNF-α) were quantified using the appropriate ELISA kits following the instructions (Proteintech, Wuhan, China).

### 2.14 Statistical Analysis

All data were expressed as the mean ± standard deviation (SD). Statistical analyses were performed using GraphPad Prism 8.0 software (GraphPad, California, USA). All trials were conducted a minimum of three times. A two-tailed paired Student’s *t* test was conducted for the two-group comparison. A one-way ANOVA test was conducted for multiple group comparison. A *P*-value less than 0.05 was considered statistically significant. *P < 0.05, **P < 0.01, ***P < 0.001, and ns indicates no statistical significance.

## 3 Results and Discussion

### 3.1 Preparation and Characterization of Cu_5.4_O NPs

Cu_5.4_O NPs were synthesized using a straightforward one-step method (Figure 1A), as previously reported.^17^ In this synthesis process, l-ascorbic acid (AA) served as both the reducing and capping agent due to its strong affinity for copper. TEM images showed that the Cu_5.4_O NPs were homogeneous and uniform in size with an average diameter of 4.47 ± 1.19 nm under dry conditions (Figure 1B–C). Compared with traditional eye drops,^20–22^ the small size of Cu_5.4_O NPs facilitated penetration through the corneal barrier. The mean hydrodynamic diameter of the Cu_5.4_O NPs was around 50.80 ± 0.37 nm (Figure 1D), and the particle distribution adhered to a Gaussian normal distribution. The zeta potential of the Cu_5.4_O NPs diminished to −19.54 mV after surface modification with AA, which may enhance the particle stability in aqueous solutions (Figure 1E). The FTIR spectra (Figure 1F) exhibited characteristic absorptions at 1467 cm^–1^ and 1635 cm^–1^, indicating the presence of AA. Additionally, peaks were observed at approximately 1460 cm^–1^ for aliphatic bending carbon–hydrogen deformations of CH_3_ and at 1673 cm^–1^ for C=O stretching vibrations. The results validated the effective production of the Cu_5.4_O NPs. To comprehensively evaluate the ROS scavenging activities of Cu_5.4_O NPs, we measured the levels of three typical ROS, including H_2_O_2_, O_2_^•–^, and •OH. Our results demonstrated that Cu_5.4_O NPs exhibited high efficiency in reducing H_2_O_2_ in a concentration-dependent manner, with approximately 80% of the total H_2_O_2_ decomposed by 1 μg mL^–1^ of Cu_5.4_O NPs (Figure 1G). However, the scavenging efficiency for O_2_^•–^ and •OH was relatively lower, with only approximately 30% of the O_2_^•–^ and 20% of the •OH decomposed under the same treatment conditions (Figure S1). Previous studies showed that Cu_5.4_O NPs catalyze the decomposition of ROS by mimicking the activity of natural enzymes. For instance, their superoxide dismutase-like activity converts O_2_^•–^ into H_2_O_2_, while their catalase-like activity further decomposes H_2_O_2_ into water and oxygen. Nevertheless, the relatively weak scavenging ability of Cu_5.4_O NPs toward •OH may be attributed to the limitations of their catalytically active sites. In summary, the successful fabrication of Cu_5.4_O NPs, characterized by their ultrasmall size and good stability, is supported by their notable antioxidative performance against H_2_O_2_.

**Figure 1:**
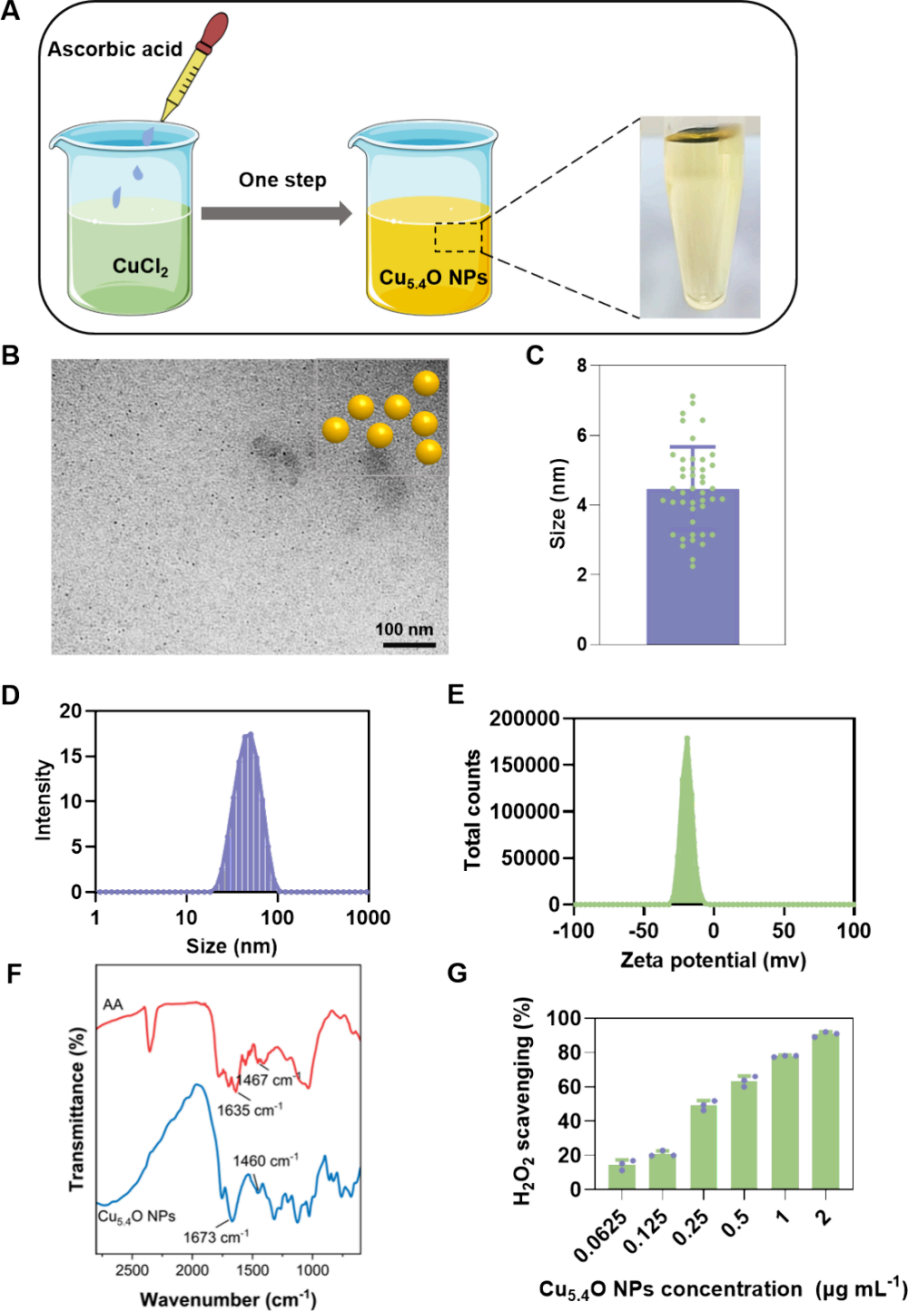
Preparation and characterization of Cu_5.4_O NPs. (A) The schematic diagram elucidates the preparation procedure for Cu_5.4_O NPs. (B) The TEM image reveals the morphology of the Cu_5.4_O NPs. The particle size is quantified in (C), and the particle size distribution is presented in (D), determined through ImageJ software analysis. (E) The zeta potential measurements indicate the stability of the Cu_5.4_O NPs. (F) The FTIR spectra compare the characteristic features of AA and Cu_5.4_O NPs. (G) The H_2_O_2_-scavenging capacities of Cu_5.4_O NPs are evaluated at various concentrations. Photograph credit: Rui Qiao, Gansu University of Chinese Medicine.

### 3.2 Cytotoxicity and Antioxidant Capacity of Cu_5.4_O NPs on HCECs *in Vitro*

Excellent nanoenzyme eye drops should have good biocompatibility. The CCK8 assay demonstrated no significant cytotoxicity in HCECs after exposure to Cu_5.4_O NPs at doses between 0 and 5 μg mL^–1^ for durations of 24 and 48 h. Notably, cell viability remained above 94% even at increasing concentrations of Cu_5.4_O NPs (0 to 2 μg mL^–1^) after 24 and 48 h of incubation (Figure 2A,B). These results indicate that Cu_5.4_O NPs exhibit excellent biocompatibility.

**Figure 2:**
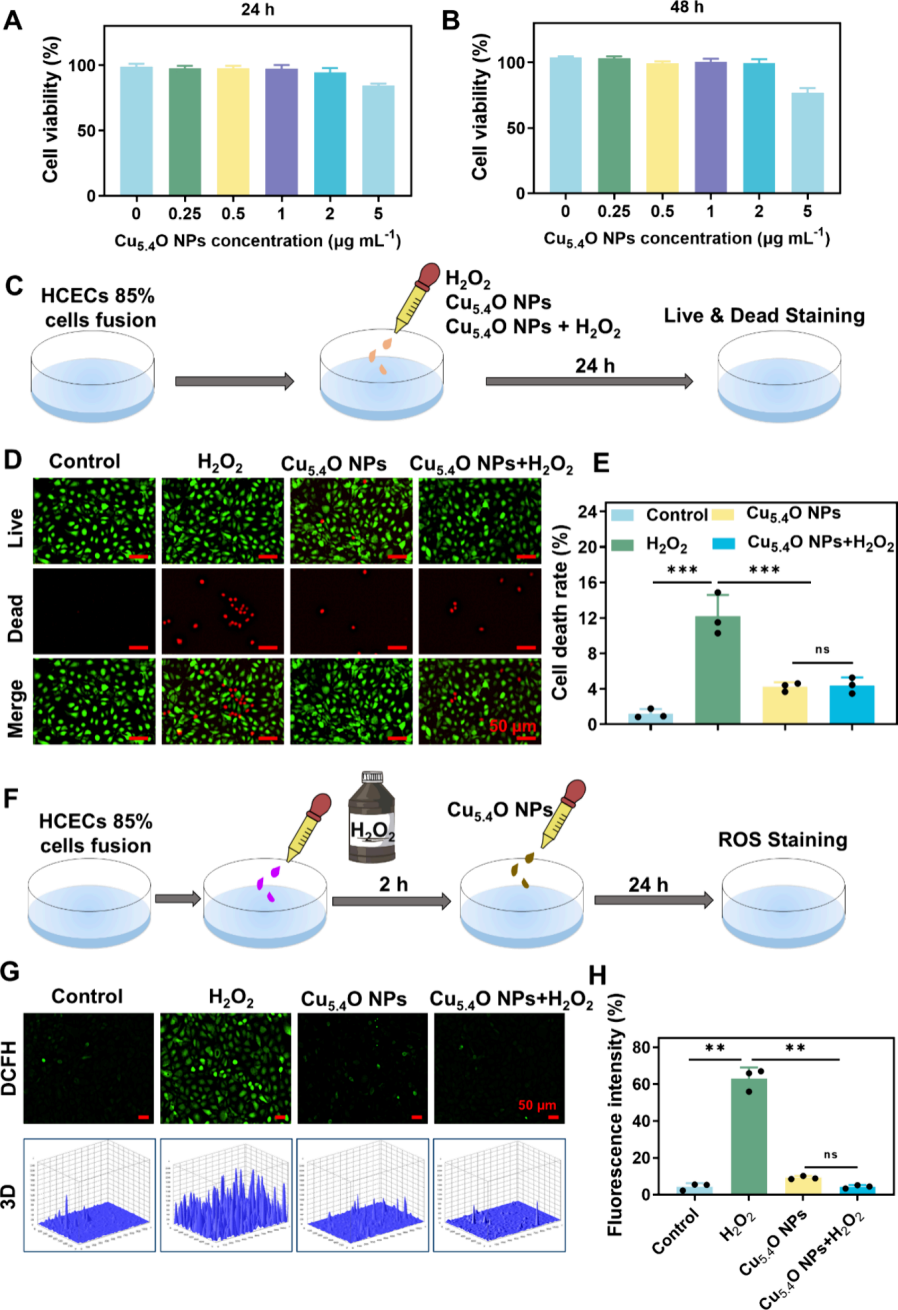
Biocompatibility and antioxidant properties of Cu_5.4_O NPs. (A, B) The viability of HCECs was assessed after 24 and 48 h of incubation with Cu_5.4_O NPs at various concentrations using the CCK-8 assay. (C, D) Representative images from Live/Dead staining of HCECs subjected to 250 μM H_2_O_2_ induction along with indicated 1 μg mL^–1^ Cu_5.4_O NP treatments are presented. Scale bar: 50 μm. (E) The percentage of dead cells, as indicated by propidium iodide (PI) staining, is shown for HCECs. (F, G) Representative images of DCF (green) fluorescence staining illustrated the levels of H_2_O_2_-induced ROS in HCECs treated with Cu_5.4_O NPs. Scale bar = 50 μm. (H) Quantitative analysis of ROS levels was conducted based on fluorescence intensity using ImageJ software. **P < 0.01, ***P < 0.001, and ns, no significance. Photograph courtesy of Liyuan Yang, Copyright 2025.

Oxidative stress results from an imbalance between free radical productions and the ability to scavenge free radicals of antioxidant defense systems.^23^ The dysregulation of oxidative stress exacerbates or induces various ocular diseases, including cataracts, keratoconus, dry eye disease, and posterior segment disorders such as proliferative vitreoretinopathy, diabetic retinopathy, age-related macular degeneration, and glaucoma.^24^ Despite the differences in their clinical expressions, treatments designed to reduce oxidative stress may postpone or avert the advancement of these significant eye diseases.^25^

Building on the H_2_O_2_-scavenging capacity of Cu_5.4_O NPs, which directly neutralize oxidative H_2_O_2_, we conducted *in vitro* assays using HCECs to assess their antioxidant capacity. For subsequent experiments, we selected a concentration of 1 μg mL^–1^ to ensure safety while evaluating the H_2_O_2_-scavenging capacity. To induce cellular oxidative damage, we used H_2_O_2_ to create oxidative stress in HCECs. Cell viability was evaluated using Live/Dead staining (Figure 2C) following 24 h of H_2_O_2_ treatment. Figure 2D,E demonstrates that the cell death rate rose to 12.21% after H_2_O_2_ treatment (*P* < 0.001), in contrast to the Control group, a phenomenon attributable to ROS-mediated oxidative damage. The cell mortality rate significantly declined to 4.40% following treatment with Cu_5.4_O NPs compared with the H_2_O_2_-treated group (*P* < 0.001). To investigate the antioxidant properties of Cu_5.4_O NPs in the context of ROS-related ocular diseases, we utilized HCECs to establish an H_2_O_2_-induced oxidative stress model. The intracellular levels of ROS in HCECs were assessed by using fluorescence staining (Figure 2F). As shown in Figure 2G,H, exposure to H_2_O_2_ significantly increased the intracellular ROS levels in HCECs. However, coincubation with Cu_5.4_O NPs resulted in a significant decrease in ROS levels compared to the H_2_O_2_ group, underscoring the cytoprotective properties of the Cu_5.4_O NPs. The findings collectively indicate that Cu_5.4_O NPs exhibit superior biocompatibility and antioxidant properties, positioning them as attractive candidates for the development of antioxidant drugs to alleviate H_2_O_2_-induced oxidative stress.

### 3.3 *In Vitro* and *in Vivo* Biocompatibility of Cu_5.4_O NPs

Hemocompatibility is a crucial factor that influences the clinical applicability of materials that come into contact with blood, making it essential for *in vivo* applicability.^26^ The hemolytic activity experiment (Figure 3A,B) indicated that different doses of Cu_5.4_O NPs, from 0.125 to 1 μg mL^–1^, produced a hemolysis ratio of below 5%. This result is below the ASTM standard hemolytic index of 5% (F756–2008),^27^ suggesting that low concentrations of Cu_5.4_O NPs demonstrate advantageous hemolytic compatibility.

**Figure 3:**
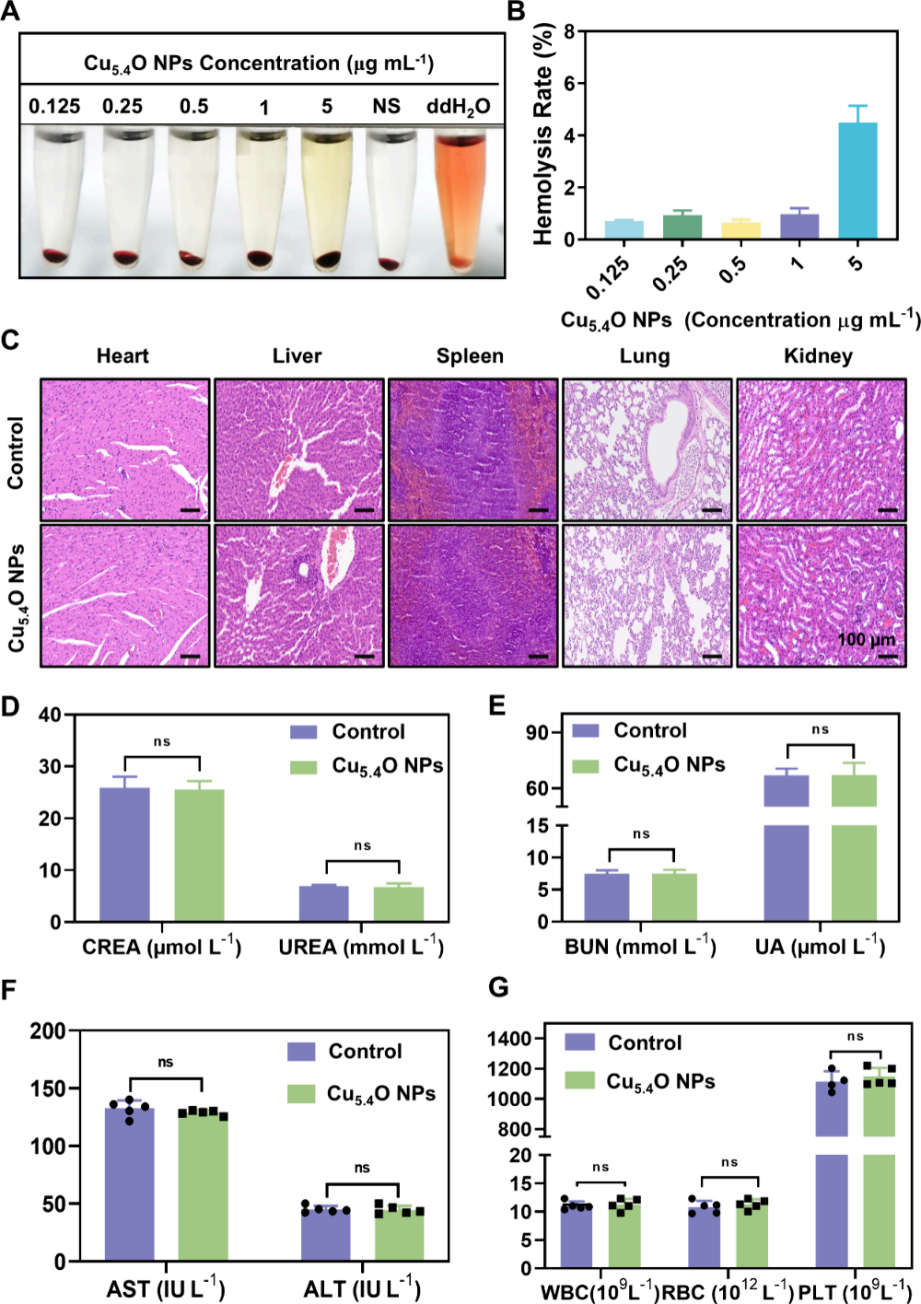
Evaluation of the biocompatibility of Cu_5.4_O NPs *in vitro* and *in vivo*. (A) Representative images from the hemolysis assay comparing the effects of the control (normal saline, NS), distilled water (ddH_2_O), and varying concentrations of Cu_5.4_O NPs on blood cells. (B) Hemolysis ratios corresponding to different concentrations of Cu_5.4_O NPs are presented. (C) *In vivo* toxicity assessment of Cu_5.4_O NPs in key organs: heart, liver, spleen, lung, and kidney—at 24 h post-treatment in normal mice (Control group) compared to those administered Cu_5.4_O NPs. (D, E) Serum levels of kidney function indicators: CREA, UREA, BUN, and UA. (F) Serum levels of liver function indicators AST and ALT. (G) Routine whole-blood parameter levels. ns, no significance. Photograph credit: Rui Qiao, Gansu University of Chinese Medicine.

Subsequently, we evaluated the biosafety, biodistribution, and excretion of Cu_5.4_O NPs to assess their potential for clinical application. Histological analysis with H&E staining indicated no observable alterations in principal organs, including the heart, liver, spleen, lung, and kidney, 24 h post-treatment with Cu_5.4_O NPs (Figure 3C). Furthermore, blood biochemistry examination revealed that the levels of renal function indicators (CREA, UREA, BUN, and UA) (Figures 3D,E) and liver function markers (AST and ALT) were similar to those in the Control group, demonstrating favorable biocompatibility in both the kidneys and liver (Figure 3F). Furthermore, a comprehensive blood panel analysis and serum biochemistry results revealed no significant discrepancies in key indices when compared to those in the Control group (Figure 3G). These findings collectively suggest that Cu_5.4_O NPs possess satisfactory biosafety. However, despite the lack of significant toxic or adverse effects observed in this study, strategies are necessary to mitigate long-term accumulation in healthy tissues and improve clearance from the body. Future research should prioritize the development of treatment strategies that can achieve precise spatial and temporal control, thereby enhancing the biosafety profile of copper-based drugs.

### 3.4 Cu_5.4_O NPs in Eye Drops Safeguard Corneas from H_2_O_2_-Induced Damage *in Vivo* to Prevent Ocular Surface Disorders

Nanoenzyme eye drops have shown remarkable therapeutic effects in corneal repair. Specifically, nanozymes can efficiently eliminate excess ROS from the ocular surface, presenting a promising approach for the prevention of dry eye illness.^16,28^ In addition, nanozyme eye drops have a longer anterior corneal residence time than traditional eye drops, reducing the need for frequent dosing.^28^

We performed *in vivo* fluorescence imaging studies using Cy5-labeled Cu_5.4_O NPs to quantitatively assess their retention time on the ocular surface and systemic metabolism. Cy5-labeled Cu_5.4_O NPs were used as eye drops in mouse models, followed by fluorescence imaging at predetermined time intervals (0, 2, 4, 6, and 12 h postadministration). As demonstrated in Figure S2, the fluorescence signal intensity analysis revealed that Cy5-labeled Cu_5.4_O NPs exhibited remarkable ocular retention, maintaining detectable fluorescence signals for more than 6 h postadministration. This extended retention time aligns with the characteristic properties of nanozymes^28^ and further supports the therapeutic potential of Cu_5.4_O NPs for ocular surface applications. Furthermore, due to their ultrasmall size (4–6 nm), Cu_5.4_O NPs were primarily metabolized through the kidneys, as evidenced by the fluorescence signal distribution in systemic tissues. These findings provide valuable insights into the pharmacokinetic behavior of Cu_5.4_O NPs, including their distribution, retention, and clearance mechanisms, which are critical for optimizing their therapeutic efficacy and safety in ocular applications.

To assess the effectiveness of Cu_5.4_O NP eye drops in preventing ROS-related corneal diseases, an *in vivo* mouse model was employed using H_2_O_2_ eye drops as a stimulus.^29^ H_2_O_2_ was administered to the central regions of the eyes to replicate external stress prior to the application of Cu_5.4_O NP eye drops (Figure 4A). After a 24 h period, the mice were euthanized, and their ocular globes were harvested for histopathological analysis to further investigate the recovery of corneal cells.

**Figure 4:**
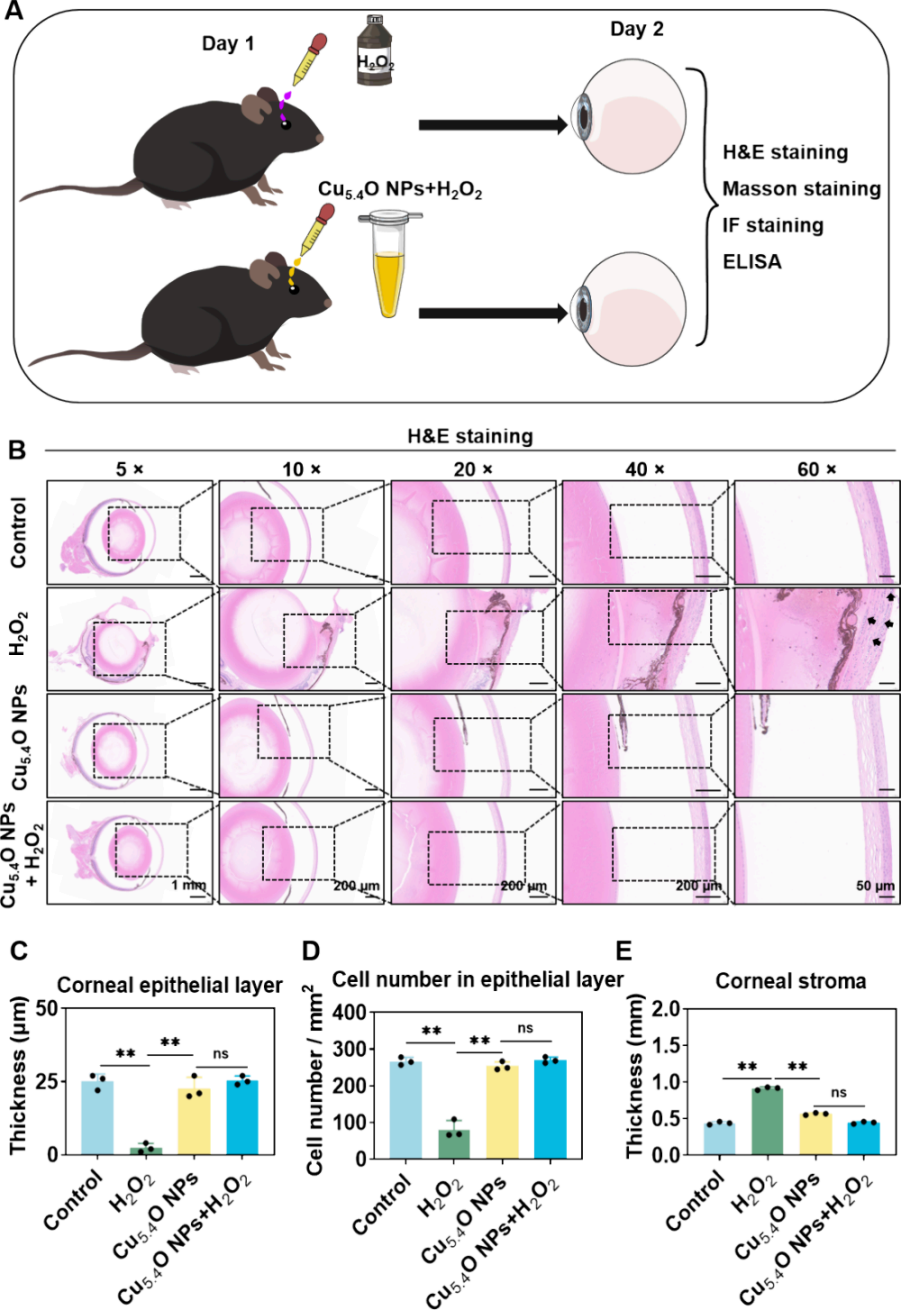
Cu_5.4_O NP eye drops alleviated corneal damage in the H_2_O_2_-induced cornea model *in vivo*. (A) Schematic representation of the H_2_O_2_-induced mouse model used to assess the protective effects of Cu_5.4_O NP eye drops. (B) Representative H&E staining images of corneal tissue from the specified groups (scale bars: 1 mm for 2×, 200 μm for 10×, 200 μm for 40×, and 50 μm for 60×). Inflammatory cells (black arrows). Statistical analysis of the thickness (C) and cell count (D) of the corneal epithelium layer. (E) Statistical analysis of the thickness of the corneal stroma. **P < 0.01 and ns, no significance. Photograph courtesy of Liyuan Yang, Copyright 2025.

H&E staining demonstrated that corneal cells were densely and orderly distributed, with no evidence of inflammatory cell infiltration in the Control group (Figure 4B).^28^ There was no significant difference in the corneal epithelial thickness, cell density in the epithelial layer, and corneal interstitial thickness between the Cu_5.4_O NP pure eye drop treatment group and the Control group, suggesting that Cu_5.4_O NP eye drops may not cause toxicity or adverse reactions (Figure 4B). However, as shown in Figure 4B for the H_2_O_2_ group, the corneal epithelial layer exhibited a reduced thickness and cell density, with markedly irregular morphology, which is indicative of epithelial injury. Additionally, the corneal stroma displayed edema, and abundant inflammatory cell infiltration was observed in the injured corneas, indicating the successful establishment of an H_2_O_2_-induced corneal damage model.^30,31^ Following treatment with Cu_5.4_O NP eye drops, the corneal tissue exhibited significant morphological and structural improvement, accompanied by a marked reduction in inflammatory cell infiltration. Quantitative analysis revealed that Cu_5.4_O NP eye drops significantly improved corneal epithelial thickness (*P* < 0.01) and corneal cell density (*P* < 0.01) compared to the H_2_O_2_-treated group (Figure 4B–D). Furthermore, stromal thickness measurements showed a significant decrease (*P* < 0.01) in the Cu_5.4_O NP eye-drop-treated group compared to H_2_O_2_ controls (Figure 4B, E), suggesting effective protection against ROS-induced stromal edema.

H_2_O_2_ has been shown to induce considerable oxidative damage to corneal stromal collagen, triggering an inflammatory response that may result in corneal melting.^29^ Masson staining revealed that in the group treated with pure Cu_5.4_O NPs the collagen fibrils displayed an arrangement similar to that found in the Control group. In contrast, the collagen fibers in the H_2_O_2_ model group were more disorganized (Figure 5A). Following treatment with Cu_5.4_O NP eye drops, the corneal structure returned to a well-organized collagen fibril arrangement and a complete lamellar structure (Figure 5A). These results suggested that Cu_5.4_O NP eye drops may reduce oxidative stress and inflammation and further prevent the corneal cells from undergoing irregular healing, thereby hindering proper corneal reconstruction.

**Figure 5:**
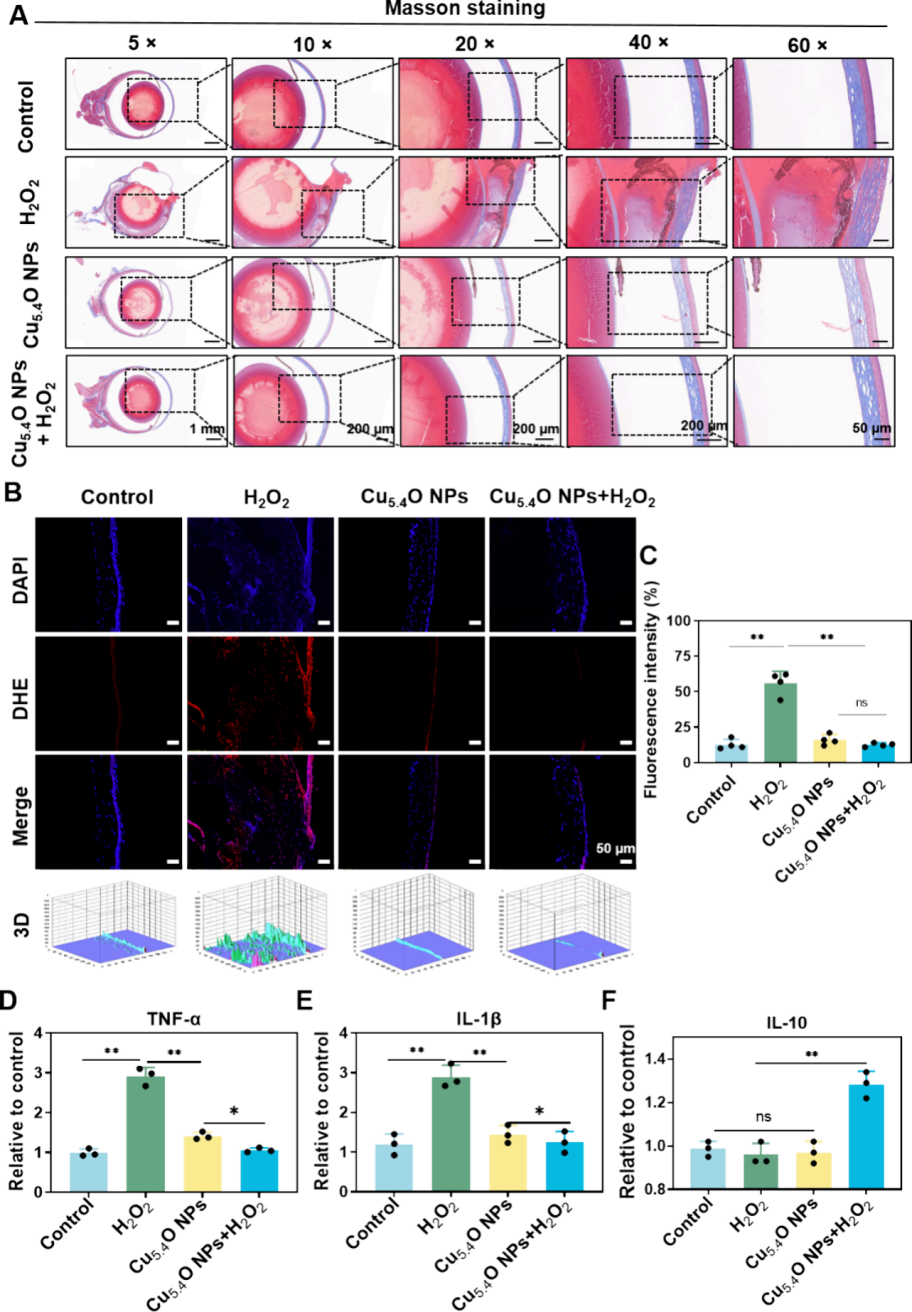
Cu_5.4_O NP eye drops scavenged corneal ROS level and reduced inflammation after cornea damage *in vivo*. (A) Representative images of Masson staining for corneal tissue from the indicated groups are shown (scale bars: 1 mm for 2×, 200 μm for 10×, 200 μm for 40×, and 50 μm for 60×). (B) DHE staining images (red) illustrate the expression levels of ROS in the corneal tissue (scale bars: 50 μm). (C) A quantitative investigation of ROS levels was performed using ImageJ to assess fluorescence intensity. The levels of TNF-α (D), IL-1β (E), and IL-10 (F) were evaluated through ELISA bioassays. *P < 0.05, **P < 0.01 and ns, no significance.

Oxidative stress, characterized by the pathological accumulation of ROS, has been mechanistically linked to the progression of diverse chronic ophthalmic pathologies including diabetic retinopathy and corneal neovascularization.^32^ Studies have demonstrated that nanoenzymes possess the ability to effectively eliminate ROS, thereby mitigating oxidative stress-induced damage to corneal cells.^28,33,34^ In this study, Cu_5.4_O NPs exhibited remarkable efficacy in scavenging ROS in both *in vitro* and cellular assays. To assess their antioxidant capacity, we evaluated the levels of ROS in the corneal tissue of mice by using DHE staining. As shown in Figure 5B,C, neither the pure Cu_5.4_O NPs or the Control group exhibited significant ROS levels. Following H_2_O_2_-induced oxidative stress modeling, a notable increase in the ROS was observed on the corneal epithelia. The Cu_5.4_O NPs + H_2_O_2_ group displayed significantly reduced ROS levels compared to the H_2_O_2_ group (*P* < 0.01), confirming the ROS scavenging capabilities of Cu_5.4_O NPs *in vivo*.

Excessive ROS can activate pathways that result in the release of inflammatory cytokines, including IL-1β and IL-6, hence contributing to ocular surface inflammation in dry eye illness.^28^ To evaluate the anti-inflammatory efficacy of Cu_5.4_O NPs, the concentrations of inflammatory cytokines TNF-α, IL-1β, and anti-inflammatory cytokines IL-10 in isolated corneas were quantified using ELISA assays. Figure 5D,E illustrate that the Cu_5.4_O NPs + H_2_O_2_ group demonstrated decreased levels of the pro-inflammatory cytokines TNF-α (*P* < 0.01) and IL-1β (*P* < 0.01) compared to the H_2_O_2_ group, indicating that Cu_5.4_O NPs may significantly contribute to the attenuation of corneal inflammation. Additionally, the Cu_5.4_O NPs + H_2_O_2_ group demonstrated a slight increase in IL-10 levels (*P* < 0.01, Figure 5F) relative to the H_2_O_2_ group. This discovery suggests that Cu_5.4_O NPs can facilitate the repair of injured ocular tissues by reducing pro-inflammatory cytokines and promoting the synthesis of anti-inflammatory cytokines. After treatment with Cu_5.4_O NPs, the ROS level was significantly reduced (*P* < 0.01, Figure 5B,C), and the expression levels of TNF-α, IL-1β, and IL-10 were restored to near-normal levels (*P* < 0.01, Figure 5D–F), demonstrating its capacity to block early pathological signals that drive subsequent inflammation. While this study focused on the early phase protective effects of Cu_5.4_O NPs, future work will extend the observation period to comprehensively assess their ability to mitigate late-phase inflammatory responses and facilitate tissue repair. These investigations will build on the foundational protective effects demonstrated here, further elucidating the therapeutic potential of Cu_5.4_O NPs in managing oxidative stress and inflammation in ocular diseases.

Here, small size Cu_5.4_O NP eye drops demonstrated efficacy in alleviating ROS-related corneal diseases through their antioxidative and anti-inflammatory properties and exhibited favorable biocompatibility for ocular use, making them appropriate for clinical application in the field of ophthalmology. While Cu_5.4_O NPs demonstrate promising ROS-scavenging capabilities and therapeutic potential for ocular diseases, certain limitations must be acknowledged. First, the long-term biosafety of Cu_5.4_O NPs requires further investigation, as excessive copper ions released via degradation may lead to cytotoxicity or unintended oxidative stress in sensitive ocular tissues.^35^ Second, the relatively narrow spectrum of ROS scavenging activity may limit their bioavailability and therapeutic efficacy. Furthermore, the possibility of off-target effects, including potential interactions with healthy cells or tissues, highlights the necessity for comprehensive *in vivo* studies to optimize dosing regimens and delivery strategies.

To address these challenges, future investigations should focus on developing advanced surface engineering approaches to enhance the biocompatibility, antioxidant efficiency, and targeted delivery of Cu_5.4_O NPs. Potential strategies may include surface modification with biocompatible polymers (e.g., PEG),^36^ proteins (e.g., albumin),^37^ or sugars (e.g., hyaluronic acid),^38^ which could improve stability and reduce immunogenicity. Additionally, innovative delivery systems such as hydrogels or contact lenses might facilitate the sustained release of Cu_5.4_O NPs, potentially enhancing their therapeutic efficacy in chronic ocular conditions like dry eye disease and age-related macular degeneration, where prolonged ROS suppression is crucial.^31^ The development of Cu_5.4_O NPs with dual enzymatic activities, mimicking both SOD and CAT, could enable more comprehensive ROS elimination. Furthermore, the incorporation of stimuli-responsive elements, such as pH-sensitive, ROS-responsive, or light-activated moieties, might allow for on-demand release of copper ions, potentially leading to the creation of intelligent drug delivery systems capable of modulating the pathological microenvironment of the ocular surface.^36,39^ These advancements hold great promise for addressing the current limitations of Cu_5.4_O NPs and unlocking their full potential as a next-generation therapeutic platform for ROS-related ocular diseases.

## 4 Conclusion

This study effectively generated ultrasmall Cu_5.4_O NPs capable of scavenging ROS, indicating their potential as innovative therapeutic agents for ROS-related ocular disorders. The Cu_5.4_O NPs are distinguished by their small particle size, elevated biocompatibility, and proficient ROS scavenging activities. Thorough *in vitro* and *in vivo* studies performed on all constituents of the eye drop formulation validated their superior biocompatibility and safety. Furthermore, in a murine H_2_O_2_-induced corneal damage model, treatment with Cu_5.4_O NP eye drops significantly protected corneal tissue by scavenging ROS and reducing inflammation. Overall, the produced ultrasmall Cu_5.4_O NPs in eye drops exhibit strong ROS scavenging capabilities and excellent biocompatibility, indicating their potential as efficient antioxidants for treating ROS-related corneal disorders.

## Supplementary Material



## Notes

Superoxide anion scavenging activity and hydroxyl radical scavenging activity of Cu_5.4_O NPs and *in vivo* fluorescence imaging distribution of Cu_5.4_O NPs (PDF)
