# Supplementary material for: Ultrasmall Copper-Based Nanozyme Eye Drops for Effective Antioxidative Therapy of Ocular Surface Diseases
Source: ACS Omega. 2025 Jun 16;10(25):26478–87. doi: 10.1021/acsomega.5c00103 (PMC12223838; doi:10.1021/acsomega.5c00103)
Supplement: Supplementary file 1 [file ao5c00103_si_001.pdf]

Supporting information

Ultrasmall Copper-Based Nanozyme Eye Drops for Effective Antioxidative Therapy of Ocular Surface Diseases

Rui Qiao[a,b, #], Liyuan Yang[c, #], Shu Zhang[c], Meiling Qian[c], Yu Lu[c], Huiling Bai[c*], Qin Liu[c,a,d*]

[a] Gansu University of Chinese Medicine, Lanzhou, Gansu, 730000, P. R. China
[b] The First People's Hospital of Lanzhou City, Lanzhou, Gansu, 730050, P. R. China
[c] Department of Ophthalmology, Gansu Provincial Hospital, Lanzhou, Gansu, 730000, P.R. China
[d] Lanzhou Aier Eye Hospital, Lanzhou, Gansu, 730099, P.R. China
[#] The author contributed equally to this work and should be considered the co-first-author

[*] Corresponding Authors:
Qin Liu E-mail: summliu@126.com
Huiling Bai E-mail: 15293112127@163.com

Supplementary Method

Superoxide anion ($O_2\cdot^-$) scavenging activity of $Cu_{5.4}O$ NPs

The $O_2\cdot^-$ scavenging activity was assessed using a superoxide anion assay kit (Nanjing Jiancheng Bioengineering Institute, Nanjing, China) following the manufacturer's instructions. Different concentrations of $Cu_{5.4}O$ NPs (0-2 μg mL^{-1}) were added to the working solution. The absorbance was measured at 550 nm using a multiple plate reader after standing for 10 min.

The scavenging activity of $O_2\cdot^-$ was evaluated utilizing a superoxide anion assay kit ((Nanjing Jiancheng Bioengineering Institute, Nanjing, China), in accordance with the manufacturer's guidelines. A series of $Cu_{5.4}O$ NPs concentrations ranging from 0 to 2 μg mL^{-1} were incorporated into the reaction mixture. Subsequently, the absorbance was quantified at a wave length of 550 nm employing a multiplate reader after a 10-minute incubation period.

Hydroxyl radical (·OH) scavenging activity of $Cu_{5.4}O$ NPs

The ·OH was generated via the classical Fenton reaction, wherein H_2O_2 reacts with ferrous ions (Fe^{2+}). The resultant ·OH radicals facilitate the oxidation of 3,3',5,5'-tetramethylbenzidine (TMB), yielding oxidized TMB that exhibits a characteristic absorption peak at 652 nm. Consequently, the concentration of residual ·OH radicals can be quantified by measuring the absorbance of oxidized TMB at 652 nm. To prepare the working solutions, a mixture containing 250 μM TMB, 2 mM H_2O_2, 1 mM $FeSO_4$, and varying concentrations of $Cu_{5.4}O$ NPs (0-2 μg mL^{-1}) in HAc/NaAc buffer (0.5 M, pH 4.5) was prepared under dark conditions and allowed to equilibrate for 5 minutes. Subsequently, TMB was introduced to each experimental group and thoroughly mixed. The absorbance of the resulting solutions at 652 nm was then measured using a multi-well plate reader to determine the extent of TMB oxidation.

Biodistribution, retention and metabolism of $Cu_{5.4}O$ NPs in vivo

The *in vivo* biodistribution, retention, and metabolism of $Cu_{5.4}O$ NPs were assessed using fluorescence imaging. Briefly, normal healthy C57BL/6 mice (6-8 weeks old, 20-25 g) were anesthetized via intraperitoneal injection of pentobarbital sodium.

Subsequently, 20 μL of Cy5-labeled $Cu_{5.4}O$ NPs (2 μg mL^{-1}) were administered as eye drops to the experimental group, while the control group received an equivalent volume of phosphate-buffered saline (PBS, 1x). Fluorescence intensity in the ocular region was monitored at predetermined time intervals (0, 2, 4, 6, and 12 hours post-administration) using an *in vivo* imaging system (IVIS Lumina XRMS Series III, PerkinElmer, Waltham, USA). To corroborate the *in vivo* observations, major organs, including eye balls, heart, liver, spleen, lung, and kidneys, were harvested for *ex vivo* fluorescence imaging of fluorescence intensities at corresponding time points following the administration of Cy5-labeled $Cu_{5.4}O$ NPs.

Supplementary Figures

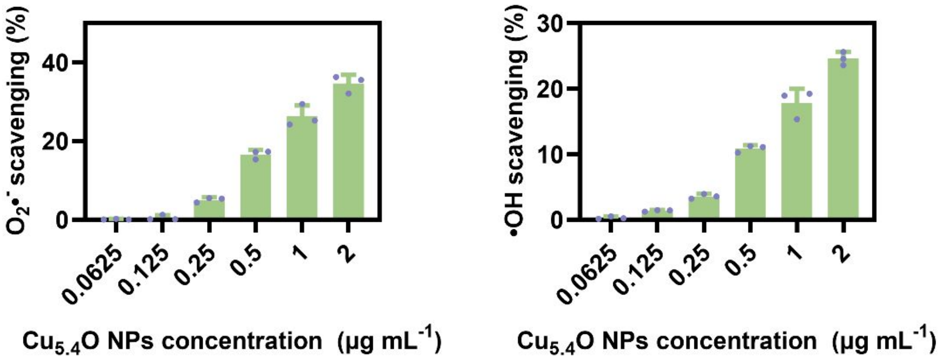

Figure S1 ROS scavenging activities of $Cu_{5.4}O$ NPs. (A)The O_2^- scavenging capacities of $Cu_{5.4}O$ NPs are evaluated at various concentrations. (B) The·OH scavenging capacities of $Cu_{5.4}O$ NPs are evaluated at various concentrations.

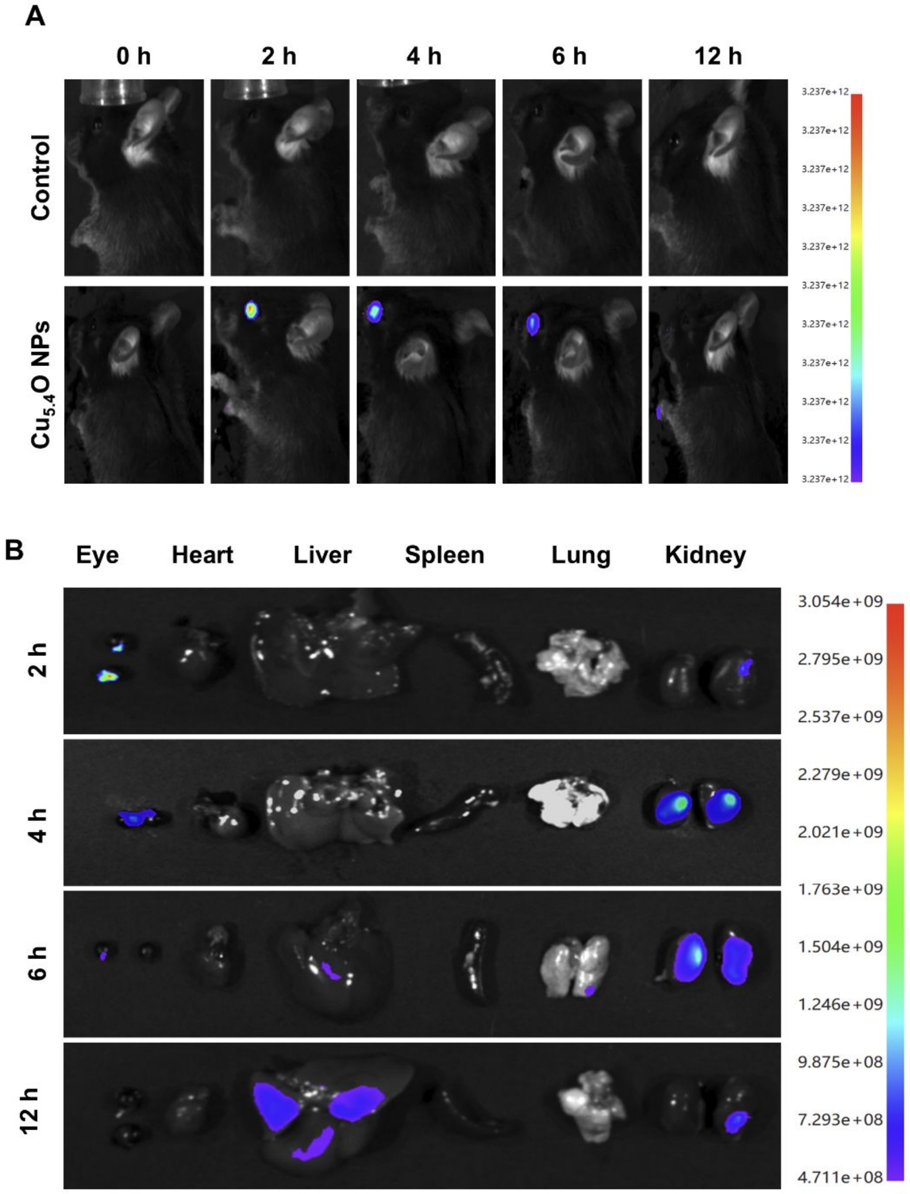

Figure S2 Distribution of $Cu_{5.4}O$ NPs *in vivo*. (A) *In vivo* imaging showed the distribution of Cy5 labeled $Cu_{5.4}O$ NPs in mouse retina after 0 h, 2 h, 4 h, 6 h and 12 h eye drops. (B) Distribution of Cy5 labeled $Cu_{5.4}O$ NPs in the eye balls, heart, liver, spleen, lung and kidney of mice at pre-determined time after eye drops.
